# Systems Genetics Approach to Biomarker Discovery: GPNMB and Heart Failure in Mice and Humans

**DOI:** 10.1534/g3.118.200655

**Published:** 2018-09-10

**Authors:** Liang-Yu Lin, Sunny Chun Chang, Jim O’Hearn, Simon T. Hui, Marcus Seldin, Pritha Gupta, Galyna Bondar, Mario Deng, Raimo Jauhiainen, Johanna Kuusisto, Markku Laakso, Janet S. Sinsheimer, Arjun Deb, Christoph Rau, Shuxun Ren, Yibin Wang, Aldons J. Lusis, Jessica J. Wang, Adriana Huertas-Vazquez

**Affiliations:** * Department of Medicine, Division of Cardiology; § Departments of Anesthesiology, Physiology, and Medicine, Cardiovascular Research Laboratories; †† Department of Human Genetics; ‡‡ Department of Biomathematics; *** Department of Microbiology, Immunology and Molecular Genetics, David Geffen School of Medicine, University of California, Los Angeles; † Division of Endocrinology and Metabolism, Department of Medicine, Taipei Veterans General Hospital, Taipei, Taiwan; ‡ Faculty of Medicine, National Yang-Ming University, Taipei, Taiwan; ** Institute of Clinical Medicine, Internal Medicine, University of Eastern Finland and Kuopio University Hospital, Kuopio, Finland; §§ Department of Biostatistics, UCLA Fielding School of Public Health, University of California, Los Angeles

**Keywords:** Biomarkers, GPNMB, Heart failure, Systems genetics, Transcriptome

## Abstract

We describe a simple bioinformatics method for biomarker discovery that is based on the analysis of global transcript levels in a population of inbred mouse strains showing variation for disease-related traits. This method has advantages such as controlled environment and accessibility to heart and plasma tissue in the preclinical selection stage. We illustrate the approach by identifying candidate heart failure (HF) biomarkers by overlaying mouse transcriptome and clinical traits from 91 Hybrid Mouse Diversity Panel (HMDP) inbred strains and human HF transcriptome from the Myocardial Applied Genomics Network (MAGNet) consortium. We found that some of the top differentially expressed genes correlated with known human HF biomarkers, such as galectin-3 and tissue inhibitor of metalloproteinase 1. Using ELISA assays, we investigated one novel candidate, Glycoprotein NMB, in a mouse model of chronic β-adrenergic stimulation by isoproterenol (ISO) induced HF. We observed significantly lower GPNMB plasma levels in the ISO model compared to the control group (p-value = 0.007). In addition, we assessed GPNMB plasma levels among 389 HF cases and controls from the METabolic Syndrome In Men (METSIM) study. Lower levels of GPNMB were also observed in patients with HF from the METSIM study compared to non-HF controls (p-value < 0.0001). In summary, we have identified several candidate biomarkers for HF using the cardiac transcriptome data in a population of mice that may be directly relevant and applicable to human populations.

Heart Failure (HF) is a complex disease characterized by a large number of pathological abnormalities including cardiac overload or injury (Braunwald 2008) and the interplay of environmental and genetic factors. In the last decade, several studies have aimed to identify clinically relevant plasma biomarkers for additional assessment of HF using different approaches. We describe a method to identify biomarker candidates using a systems genetics approach, in which a diverse population of individuals is examined for traits of interest as well as high-throughput molecular phenotypes, such as global transcript levels in relevant tissues. Systems genetics approaches can of course be applied to human populations, but mice offer the advantage of avoiding confounders such as disease heterogeneity and differences in environmental factors (Lusis, Seldin *et al.* 2016). Among existing inbred strains, genetic and phenotypic diversity is as great as that observed in the human population and unlike humans, mice can be subjected to experimental breeding and tissue collection for detailed phenotyping and transcriptomic analyses (Attie, Churchill *et al.* 2017). Hundreds of human disease models have been developed in mice and nearly all of these appear to be affected by the genetic background of the mouse (Riordan and Nadeau 2017).

We have developed a systems genetics resource termed the Hybrid Mouse Diversity Panel (HMDP), where the inbred mice were chosen for diversity. They have been maintained under a variety of environmental conditions, typed for various clinical traits, and subjected to global transcriptomic profiling of relevant tissues (Lusis, Seldin *et al.* 2016). We describe a study for one trait previously investigated in the HMDP, heart, failure (HF). We show that the list of genes, whose transcript levels in heart correlate most strongly with HF traits, includes known biomarkers of human HF. We investigate a novel potential HF biomarker, Glycoprotein NMB (GPNMB), in both mice and humans. GPNMB is a type 1 transmembrane protein also known as osteoactivin (Selim 2009) that has been recently involved in inflammation, fibrosis and myocardial remodeling (Järve *et al.* 2017).

## Materials And Methods

### Analysis of Hybrid Mouse Diversity Panel (HMDP) cardiac transcriptome data

The differential expression of cardiac transcriptome data from 91 inbred strains of the Heart Failure-HMDP study has been published previously (Wang, Rau *et al.* 2016). We performed correlation analysis of the change in left ventricular internal dimension (LVIDd) from baseline to week 3 of isoproterenol and cardiac transcript levels at week 3 of isoproterenol.

### Analysis of GPNMB transcript level in the human Myocardial Applied Genomics Network (MAGNet) study

In order to confirm the upregulation of *GPNMB* in humans during HF, we examined available human cardiac transcriptome data from the MAGNet consortium. The MAGNet consortium has collected and evaluated the cardiac transcriptome using microarrays for 313 subjects at the time of heart transplant or explant [95 individuals with ischemic cardiomyopathy (ICM), 82 with dilated cardiomyopathy (DCM), and 136 non-heart failure (NF) unused donors (Das, Morley *et al.* 2015, Liu, Morley *et al.* 2015). RNA-Seq and microarray data have been deposited in the Gene Expression Omnibus (GEO) Database (Accession number GSE57345). Differential gene expression analysis was performed using GEO2R available on the GEO website.

### Mouse Models of Heart Failure

We assessed circulating GPNMB levels in 2 well-established mouse HF models: The pressure overload by transverse aortic constriction (TAC) and the chronic β-adrenergic stimulation by continuous isoproterenol (ISO)-induced cardiac hypertrophy. For the TAC model, mice were divided into TAC or sham surgery groups. Sham mice received a midsternal incision to expose only the transverse aorta. For the ISO model, mice were divided into control and ISO treatment groups. ISO was administered via an intraperitoneal minipump that delivers a continuous infusion of 30 mg/kg/day for 21 days. The ISO dose was determinated according to previously published data and our HMDP study (Oudit, Crackower *et al.* 2003, Berthonneche, Peter *et al.* 2009, Galindo, Skinner *et al.* 2009, Wang, Rau *et al.* 2016). Both HF models were performed in 10-week-old female C57BL/6J mice.

Plasma samples were collected by retro-orbital puncture at the time of euthanasia, which was at 4 weeks after intervention for TAC mice (n = 6) and at 3 weeks after infusion pump implantation for ISO mice (n = 10). Upon conclusion of the experiments, animals were Sacrifice and the hearts were removed. The UCLA Institutional Animal Care and Use Committee (IACUC) approved all animal studies.

### Echocardiography

Echocardiograms were performed using the Vevo 2100 ultrasound system (VisualSonics, Inc., Toronto, ON, Canada). A parasternal long-axis B-mode image was obtained. The maximal long-axis of the LV was positioned perpendicular to the ultrasound beam. A 90° rotation of the ultrasound probe at the papillary muscle level was performed to obtain a parasternal short-axis view of the LV. A M-mode image to document LV dimensions was captured and saved for analysis using the Vevo 2100 cardiac analysis package. Baseline echocardiograms were performed on all of the mice. In the isoproterenol cohort, final echocardiograms were performed for control and isoproterenol-treated mice at week 3 of the experiment. In the TAC group, final echocardiograms were performed for control and TAC-treated mice at week 4 of the experiment. To ensure adequate sedation while minimizing the effects of inhaled isoflurane on loading conditions, heart rate, cardiac structure and function, we minimized induction and maintenance doses of isoflurane at or below 1.25% and 1%, respectively, while closely monitoring for HR < 475 bpm as a sign of deep sedation and adjusting isoflurane dosage as needed (Wang, Rau *et al.* 2016).

### Western blot analysis of GPNMB in heart tissues of mice

Proteins from the heart tissue of ISO treated, TAC mice, and control mice were harvested in buffer (50mM HEPES [pH 7.4], 150mM NaCl, 1% NP-40, 1mM EDTA, 1mM EGTA, 1mM glycerophosphate, 2.5mM sodium pyrophosphate 1mM Na3VO4, 20mM NaF, 1 mM phenylmethylsulfonyl fluoride, 1 μg/mL of aprotinin, leupeptin, and pepstatin). Equal amounts of protein were separated on 4–12% Bis-Tris gels (Invitrogen, Carlsbad, CA) using an electroblotting apparatus (Bio-Rad Laboratories, Hercules, CA) and transferred onto a nitrocellulose blot (Amersham, GE Healthcare). The blot was probed with the indicated primary antibodies using the polyclonal anti-GPNMB (R&D Systems, Minneapolis, MN) and anti-GAPDH (Invitrogen, Carlsbad, CA). Protein signals were detected using HRP conjugated secondary antibodies (Cell Signaling Technologies) and enhanced chemiluminescence (ECL) western blotting detection regents (Amersham, GE Healthcare).

### Cross-sectional study of the METabolic Syndrome In Men (METSIM) cohort

The METSIM study is comprised of 10,197 Finnish men recruited between age 45 to 74 years (mean ± SD = 58 ± 7 years) by random sampling from the population register of Kuopio, Eastern Finland. The METSIM study and its methods have been described in detail elsewhere (Stancakova, Javorsky *et al.* 2009, Laakso, Kuusisto *et al.* 2017). The METSIM HF cases were identified by screening medical records for HF diagnostic codes and by querying the Finnish medication reimbursement database for HF medications. A total of 119 subjects with HF were identified and 270 control subjects with no previous diagnosis of HF or current clinical or biochemical indication of cardiovascular diseases or other chronic disease including chronic kidney disease and end stage renal disease patients were determined to be controls. The study was approved by the Ethics Committee of the University of Eastern Finland and Kuopio University Hospital.

### GPNMB measurements in mice and humans

Plasma GPNMB levels in mice and human samples were assayed using commercial enzyme-linked immunosorbent assay kits (R&D systems, Minneapolis, MN) (Catalogue numbers: DY2330 and DY2550, respectively).

### Statistical analysis

The *t*-test statistic was used to examine differences between HF and control plasma GPNMB protein levels in mice. The p-value threshold of < 0.05 was considered statistically significant. Clinical characteristics of HF cases and non-HF controls were compared using *t*-tests for continuous variables and Fisher’s exact tests for categorical variables. A Spearman rank correlation test was used to assess the correlation between GPNMB and proBNP. The associations between GPNMB and HF were investigated by univariate and multivariate logistic regression models using age, BMI, hypertension, diabetes, eGFR and LDL-C levels as covariates to control for potential confounders. These covariates were chosen based on data from previous reports (Wang, Larson *et al.* 2002, Barasch, Gottdiener *et al.* 2009, Duprez, Gross *et al.* 2018) and clinical data available from the METSIM study. All the statistical analyses were performed with the SPSS statistical software package.

### Data availability

The HMDP cardiac transcriptome data are available at the Gene Expression Omnibus (GEO) online database by the accession GSE48760 (Wang, Rau *et al.* 2016). The complete correlation data of cardiac transcripts with ISO-induced left ventricular dilation is presented in Supplemental Table 1. Supplemental table 2 includes unidentified clinical data of all METSIM subjects included in this study. Supplemental material available at Figshare: https://doi.org/10.6084/m9.figshare.7069673.

## Results

### Selection of GPNMB as a candidate biomarker for HF

The list of transcripts that were most perturbed in terms of fold change by isoproterenol, including GPNMB (FC = 1.6, p-value = 1.92X10^−12^) are shown in Table 1. In addition, we ranked transcripts by the magnitude of correlation with left ventricular dilation, a clinical trait we used as a surrogate marker of adverse cardiac remodeling (Table 2). Of interest, the top correlated transcripts with left ventricular dilation corresponded to genes involved in collagen synthesis and degradation (*Col6a1* (Luther, Thodeti *et al.* 2012), *Col5a1* (Roulet, Ruggiero *et al.* 2007), *Fbn1* (Fedak, de Sa *et al.* 2003)), remodeling of the heart and arterial calcification (*Dtr*, *Spp1* (Peacock, Huk *et al.* 2011), *Enpp1*(Pillai, Li *et al.* 2017)), extracellular matrix synthesis and degradation (*Ctsk* (Hua, Xu *et al.* 2013), *Sparc* (Bradshaw 2009, Toba, de Castro Bras *et al.* 2016) and *Mfap5* (Vaittinen, Kolehmainen *et al.* 2015)).

**Table 1:** Top differentially regulated genes in the ISO *vs.* control cardiac transcriptome

PROBE_ID	SYMBOL	logFC	AveExpr	p-value
ILMN_3103896	Timp1	2.04	8.75	5.21E-26
ILMN_2769918	Timp1	2.03	8.69	1.08E-25
ILMN_1246800	Serpina3n	2.01	9.15	4.24E-24
ILMN_2654624	AI593442	1.88	8.00	7.90E-28
ILMN_1223317	Lgals3	1.82	8.65	9.73E-26
ILMN_2648669	Gpnmb	1.61	6.97	1.92E-12
ILMN_1239726	Snai3	−1.51	7.03	3.60E-26
ILMN_2690603	Spp1	1.31	5.36	4.40E-11
ILMN_2997494	Lox	1.30	6.91	2.97E-17
ILMN_1218235	Gnb3	−1.19	6.81	1.35E-25
ILMN_1232261	Catnal1	1.18	11.00	7.77E-29
ILMN_1226472	Retnla	−1.17	6.51	6.30E-17
ILMN_2975345	Cdo1	1.17	7.84	5.48E-11
ILMN_3127595	BC020188	1.14	6.55	4.28E-21
ILMN_2666312	BC025833	−1.09	8.77	1.90E-21
ILMN_2844820	Angptl7	1.09	7.80	2.58E-07
ILMN_2625279	Pacrg	1.09	6.78	8.73E-28
ILMN_2950622	Arhgdig	1.07	6.09	2.23E-28
ILMN_3091003	Ms4a7	1.05	7.41	1.14E-22
ILMN_1238886	Ccl8	1.04	6.34	1.82E-11
ILMN_1222196	2310007A19Rik	1.03	6.82	1.20E-23
ILMN_2968211	Lgals4	−1.03	9.78	1.15E-14

LogFC: Log fold change; AveExpr: Average expression; adj. P Val: adjusted p-value. The AveExpr is the ordinary arithmetic average of the log2-expression values for the probe, across all arrays.

**Table 2:** Top correlated transcripts with isoproterenol-induced left ventricular dilation

ilmn_id	symbol	cor	p-value
ILMN_2698449	Dtr	0.433	2.6E-05
ILMN_2768087	Col6a1	0.429	3.1E-05
ILMN_2636424	Itgbl1	0.414	6.2E-05
ILMN_2818294	Srpx2	0.410	7.3E-05
ILMN_2883952	1810015A11Rik	0.410	7.3E-05
ILMN_2887408	Galr3	0.406	8.6E-05
ILMN_2597831	Cacna1c	−0.403	9.9E-05
ILMN_2748402	Col5a1	0.398	1.2E-04
ILMN_2603958	9130427A09Rik	0.391	1.6E-04
ILMN_2721149	Arl11	0.389	1.8E-04
ILMN_2946873	D030070L09Rik	0.388	1.9E-04
ILMN_2638256	Tex16	−0.379	2.7E-04
ILMN_1259388	Col6a1	0.378	2.8E-04
ILMN_2711163	Ctsk	0.378	2.8E-04
ILMN_2811421	Matk	0.377	2.9E-04
ILMN_2782964	Enpp1	0.376	3.1E-04
ILMN_2664660	Aldh5a1	−0.375	3.2E-04
ILMN_2690603	Spp1	0.374	3.4E-04
ILMN_3136561	Sparc	0.373	3.4E-04
ILMN_1232884	Sphk1	0.371	3.7E-04
ILMN_2750201	1700023I07Rik	0.370	3.8E-04
ILMN_2975345	Cdo1	−0.370	3.8E-04
ILMN_2641956	Nab2	−0.370	3.9E-04
ILMN_2833163	BC064033	0.369	4.0E-04
ILMN_2613601	2010001M09Rik	−0.367	4.3E-04
ILMN_1231851	Enpp1	0.365	4.6E-04
ILMN_2953515	Aldh3b1	0.365	4.7E-04
ILMN_1223552	Fbn1	0.365	4.8E-04
ILMN_2645526	Abcc8	−0.363	5.1E-04
ILMN_2614655	Gpnmb	0.363	5.2E-04
ILMN_1214571	Cd109	0.361	5.5E-04
ILMN_1225835	Mfap5	0.360	5.6E-04
ILMN_2702704	Ndufv1	−0.359	5.9E-04
ILMN_2725484	Padi4	0.359	6.0E-04
ILMN_2691951	Polydom	0.358	6.2E-04
ILMN_1221611	Pitpn	0.357	6.3E-04
ILMN_1228485	Csnk2a2	−0.356	6.6E-04
ILMN_2838317	Pqlc3	0.356	6.6E-04
ILMN_1221800	Gabpa	−0.356	6.6E-04
ILMN_2646254	1700102P08Rik	−0.356	6.6E-04
ILMN_3022719	Wiz	−0.355	6.8E-04
ILMN_2453695	Urod	−0.353	7.5E-04
ILMN_2837100	Gm128	0.352	7.6E-04
ILMN_3116885	Gpr137b	0.352	7.7E-04
ILMN_2487358	Eif3s6	0.351	8.1E-04
ILMN_2671755	Ceecam1	0.351	8.1E-04
ILMN_2492500	Zfhx1a	0.351	8.1E-04

We examined cardiac expression of *Nppb*, *Timp1* and *Lgals3*, which are transcripts encoding three well-known heart failure plasma biomarkers brain natriuretic peptide (BNP), tissue inhibitor of metalloproteinase 1 (TIMP1), and galectin-3 (de Boer, Voors *et al.* 2009, Goldbergova, Parenica *et al.* 2012, Ho, Liu *et al.* 2012). *Timp1* and *Lgals3* increased by 3.5- to 4 fold with isoproterenol treatment (Table 1). Both transcripts were also positively correlated with isoproterenol-induced left ventricular dilation (Table 2; *Timp1*: r = 0.25, p-value = 0.02; *Lgals3*: r = 0.29, p-value = 0.006). Interestingly, although *Nppb* level was positively correlated with left ventricular dilation (r = 0.26, p-value = 0.01), *Nppb* level was not significant altered by isoproterenol.

Next, we performed differential gene expression analysis of microarray-based transcriptome data deposited in the Gene Expression Omnibus (GEO) database (GSE57345 GPL9052) from the MAGNet consortium human cardiac tissue collection using GEO2R. MAGNet consortium collected and evaluated the cardiac transcriptome by microarray at the time of heart transplant or explant (Das, Morley *et al.* 2015, Liu, Morley *et al.* 2015). *TIMP1* and *LGALS3* were significantly differentially expressed in the MAGNet study (LogFC = -0.69, p-value = 6.32 × 10^−17^ and logFC = 0.17, p-value = 8.30 × 10^−6^, respectively). As observed in mice, *NPPB* levels were not differentially expressed between HF cases and control subjects (p-value = 0.32).

We overlaid the top differentially expressed (Table 1) and correlated (Table 2) lists from the heart failure HMDP to identify novel candidate transcripts that were both differentially regulated by isoproterenol and correlated significantly to left ventricular dilation. *Cdo1* and *Gpnmb* fit both criteria. While *Cdo1* was negatively correlated with left ventricular dilation (probe ID = ILMN_2975345, r= -0.37, p-value = 3.8 × 10^−4^), *Gpnmb* was positively correlated with left ventricular dilation (probe ID = ILMN_2614655, r= 0.36, p-value = 5.2 × 10^−4^). *Gpnmb* encodes a transmembrane protein expressed in macrophages and has an ectodomain that is shed by its regulatory protein ADAM10 to the extracellular compartment. We chose to follow up on GPNMB after confirming that *GPNMB* transcript levels were similarly upregulated in failing *vs.* non-failing hearts by 1.2 fold (p-value= 2.9 × 10^−6^) in subjects from the MAGNet cohort.

### GPNMB levels in two mouse models for HF

**Figure 1:**
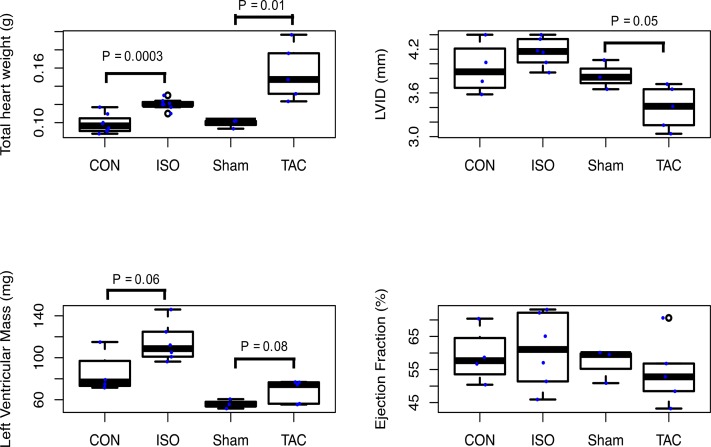
Isoproterenol and transverse aortic constriction induced cardiac remodeling characteristics among C57BL/6J mice. LVID denotes left ventricular internal dimension during diastole. CON denotes control. ISO denotes isoproterenol infusion at 30 mg/kg/day for 21 days. TAC denotes transverse aortic constriction for 28 days.

**Figure 2:**
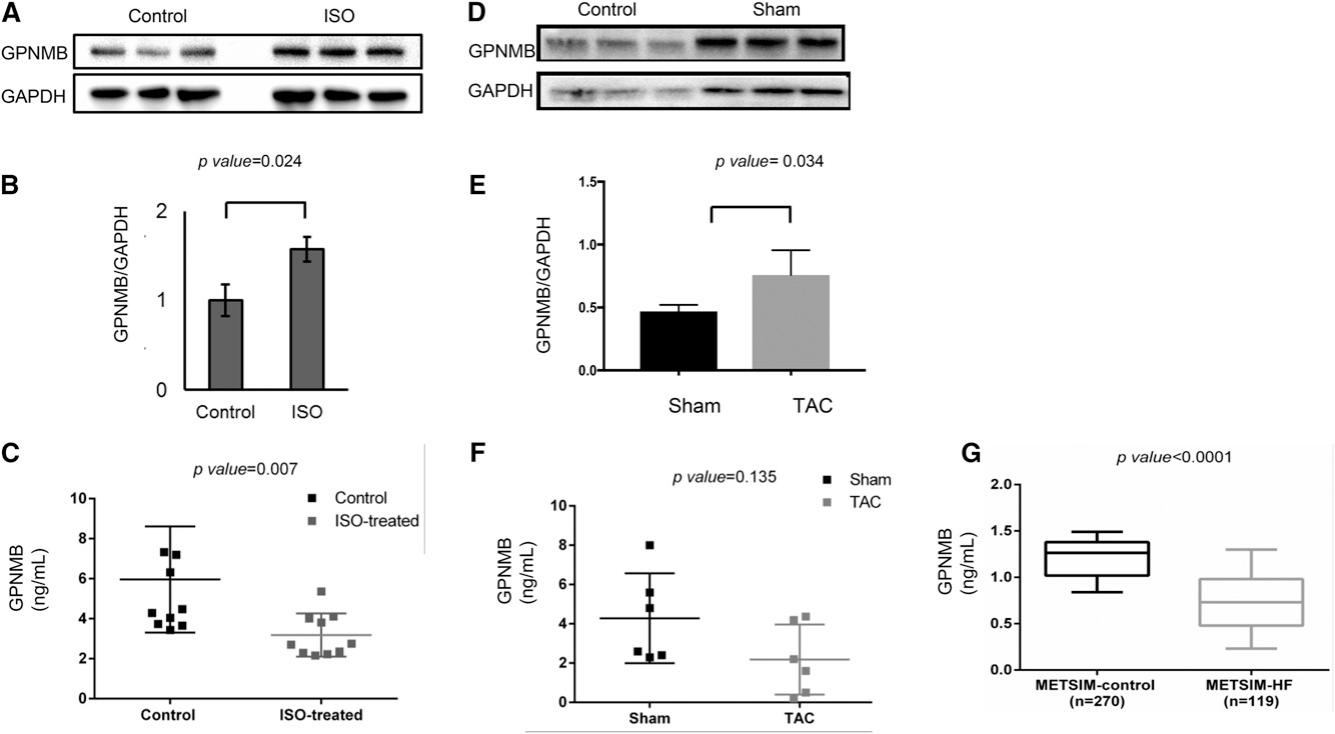
GPNMB levels in Isoproterenol, transverse aortic constriction (TAC) heart failure mouse models and in patients with heart failure. A. Western blot analysis of GPNMB expression in C57BL/6J mouse heart lysates after ISO treatment. B. Graphic representation of Western blot analysis for the ISO model. C. GPNMB plasma levels comparison between control and ISO-treated mice. For the ISO model, mice were anesthetized with intraperitoneal ketamine as a surgical anesthetic agent, and osmotic minipumps were implanted subcutaneously. D. Western blot analysis of GPNMB expression in C57BL/6J mouse heart lysates after TAC surgery. GAPDH was used as housekeeping control. * p-value < 0.05 for student’s *t*-test. E. Graphic representation of Western blot analysis for the TAC model. F. GPNMB plasma levels comparison between Sham and TAC. For the TAC model, midsternal incision was made to expose transverse aorta between truncus anonymous and the left carotid artery. G. GPNMB levels in HF patients and controls from the METSIM study.

To confirm the protein levels of GPNMB in heart failure, we used two widely accepted modes of cardiac injury, isoproterenol (ISO) and transverse aortic constriction (TAC), to induce a heart failure-like state in mice. Both models lead to cardiac hypertrophy as measured by heart weight at sacrifice and left ventricular mass estimates by echocardiography (Figure 1). Consistent with our observation in the cardiac transcriptome, GPNMB protein expression in the heart was increased in mice treated with ISO as compared to controls (Figure 2A and 2B). Similarly, GPNMB protein level in the TAC hearts also showed a significant increase as compared with sham animals (Figure 2C and 2D), indicating that there is increased GPNMB cardiac expression in two different HF mouse models.

Furthermore, we measured plasma GPNMB protein levels in the ISO and TAC models. In the ISO model, after 3 weeks of continuous infusion of ISO, plasma levels of GPNMB were lower than in the control group (5.96 ± 2.66 ng/mL in control *vs.* 3.18 ± 1.08 in ISO, p-value = 0.007) (Figure 2C). Although the plasma GPNMB levels in the TAC model compared with the sham surgery group at 4 weeks after surgery were not statistically significantly different due to small sample sizes, there was a trend toward decreased GPNMB levels in the TAC mice (4.19 ± 2.33 ng/mL in Sham *vs.* 2.22 ± 1.80 ng/ml in TAC, p-value = 0.13) (Figure 2F).

### GPNMB levels in human HF From the METSIM study

**Figure 3:**
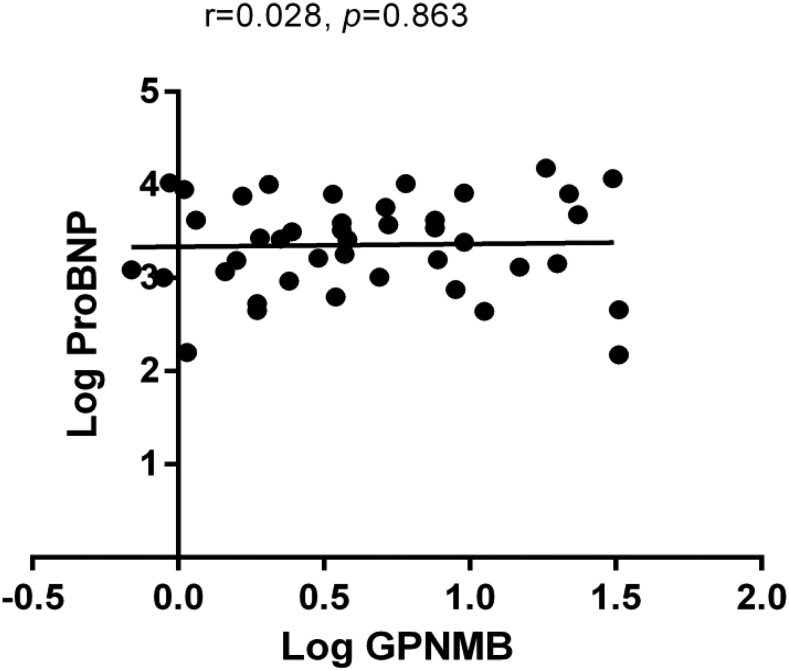
Correlation between GPNMB, proBNP levels in the METSIM study. The total number of subjects with proBNP and GPNMB levels was n = 42.

We measured plasma GPNMB levels in 119 HF subjects and 270 non-HF controls from the METSIM study. Patients’ baseline characteristics are listed in Supplemental Table 3. The distribution of plasma GPNMB did not reveal normality in both control and HF groups, thus we used log GPNMB in these analyses. As observed in the ISO mice, there were significantly lower plasma GPNMB levels in patients with HF compared with non-HF controls (GPNMB 1.20 ± 0.26 ng/mL in control *vs.* 0.74 ± 0.40 ng/mL in heart failure, p-value < 0.0001) (Figure 2G). To prevent bias due to an age difference between HF cases and controls, we performed sensitivity analysis confirming that our results were not affected by age differences between the groups (p-value < 0.001). GPNMB level, age, BMI, history of HTN and DM, eGFR and LDL-C were significantly associated with HF (Table 3) and were included in the multivariate analysis. The association between GPNMB and HF remained significant in the multivariate analyses (OR = 0.86 [0.82-0.90], p-value < 0.001). In a subset of HF cases, where proBNP levels were available, GPNMB and proBNP were found to be independent (r =0.028, p-value =0.863), suggesting that measurement of GPNMB in plasma of HF patients may provide additional prognostic value or reflect different clinical or biological states from those associated with proBNP elevation (Figure 3).

**Table 3:** Univariate and multivariate logistic regression analysis of the variables associated with the presence of heart failure

	Univariate analysis		Multivariate analysis	
Variables	OR(95% CI)	*P-value*	OR(95% CI)	*P-value*
GPNMB, ng/ml	0.865 (0.834-0.896)	<0.001	0.863 (0.824-0.904)	<0.001
Age, years	1.306 (1.233-1.384)	<0.001	1.277 (1.182-1.379)	<0.001
Body mass index kg/m^2^	1.188 (1.124-1.256)	<0.001	1.142 (1.057-1.233)	0.001
Hypertension	6.173 (3.703-10.309)	<0.001	2.922 (1.286-6.643)	0.010
Diabetes mellitus	13.699 (6.536-28.571)	<0.001	6.711 (2.128-21.277)	0.001
eGFR, mL/min/1.73 m^2^	0.975 (0.960-0.989)	0.001	0.994 (0.971-1.017)	0.603
LDL-c, mg/Dl	0.972 (0.965-0.980)	<0.001	0.991 (0.980-1.002)	0.097

GPNMB: glycoprotein non-metastatic melanoma protein B; eGFR: estimated glomerular filtration rate; LDL-c: low density lipoprotein cholesterol; OR: Odds ratio; CI: confidence interval.

## Discussion

In the present study, we analyzed global cardiac transcriptomic data from the Heart Failure-HMDP study as a strategy to identify novel plasma biomarkers for heart failure. We found that cardiac transcripts of established HF plasma biomarkers, including TIMP1 and galectin-3, were differentially expressed in ISO-treated mouse hearts and correlated with left ventricular dilation compared to the control group. We identified *Gpnmb* as an attractive candidate based on similar properties and confirmed its upregulation in the MAGNet human heart failure transcriptome collection. Next, we confirmed the upregulation of GPNMB protein levels in ISO and TAC mice. Subsequently, we examined plasma GPNMB levels in mice treated with ISO and TAC. We found significantly lower levels of circulating GPNMB in the ISO model and a trend toward decrease in the TAC model. This was a surprising finding not explained by a known mechanism. We also investigated whether lower levels of circulating GPNMB were found in human HF patients. Similar to our observation in mice, circulating plasma GPNMB levels were also lower in patients with HF from the METSIM study compared to the control group.

GPNMB has been shown to play a role in promoting tissue regeneration after muscle, kidney, liver and cerebral ischemia reperfusion injury by regulation of immune/inflammatory responses and suppressing fibrosis (Abe, Uto *et al.* 2007, Furochi *et al.* 2007a,b, Nakano, Suzuki *et al.* 2014, Nagahara, Shimazawa *et al.* 2015). Previous studies using different cardiac injury models have shown that cardiac tissue levels of GPNMB generally increased in response to stress. These include the desmin knockout mouse model (Psarras, Mavroidis *et al.* 2012), the Theiler’s murine encephalomyelitis virus-induced acute viral myocarditis model (Omura, Kawai *et al.* 2014), and the myocardial infarction rat and mouse models (Järve *et al.* 2017). In the myocardial infarction model, GPNMB mRNA transcript was up-regulated 17-fold in the peri-infarct (PI) area in the rat and 300-fold in the mouse at 24 hr and 7 days after myocardial infarction, respectively. Approximately 50% of the CD68+ macrophages expressed GPNMB (Järve *et al.* 2017). Similar to these publications, we observed an upregulation of *Gpnmb* by isoproterenol on average across the HMDP mouse strains, in the MAGNet human heart failure transcriptome data and two different cardiac injury models, isoproterenol and transverse aortic constriction, in C57BL/6J mice.

The exact mechanism by which GPNMB exerts its effect on the heart is not clear. Increased GPNMB expression is seen following injury in multiple organs including the heart (Järve *et al.* 2017) and kidney (Zhou, Zhuo *et al.* 2017) and GPNMB could be playing organ specific roles in wound healing. In this regard, it was observed in a study comparing GPNMB-deficient DBA/2J mice and their coisogenic DBA/2J-GPNMB+ relatives, that GPNMB appeared to confer increased risk of adverse ventricular modeling with left ventricular dilation and a decrease in fractional shortening after myocardial infarction (Järve *et al.* 2017). Because GPNMB has been implicated in endothelial adhesion and transendothelial migration (Shikano, Bonkobara *et al.* 2001), Jarve *et al.* postulated that GPNMB-deficiency may impair *trans*-endothelial migration of monocytes from blood to cardiac tissue. Indeed, elevated numbers of monocytes with the proinflammatory Ly6C^high^ phenotype were identified in the blood and bone marrow of GPNMB-deficient mice (Järve *et al.* 2017). In contrast, the same adverse impact of GPNMB on cardiac remodeling was not observed after isoproterenol (Järve *et al.* 2017). This could be related to the fact that isoproterenol infusion is associated with decreased inflammatory infiltrate compared to an acute injury such as myocardial infarction that is associated with an intense inflammatory infiltrate in the heart. Moreover, previous studies have suggested that GPNMB serves as an inflammatory stop signal in HF that inhibits the activation of T lymphocytes by binding syndecan 4 (Chung, Sato *et al.* 2007), a proteoglycan that is up-regulated in chronic HF (Takahashi, Negishi *et al.* 2011) and has been previously shown to adversely influence cardiac remodeling (Kojima, Takagi *et al.* 2001). If true, increased consumption of GPNMB or lower circulating levels of GPNMB could be indicative of more severe HF. Taken together, whether GPNMB expression is deleterious to cardiac remodeling may depend upon the mode of injury, type of inflammatory response present, and local cellular expression *vs.* circulatory levels of GPNMB ectodomain. Additional studies using different cardiac injury models, examining inflammatory response and sites of GPNMB action are needed to fully delineate GPNMB’s relationship with cardiac injury and remodeling.

The observation of directionally opposite changes in biomarker abundance in tissue *vs.* plasma is especially intriguing. GPNMB, also known as osteoactivin, is a highly-glycosylated type I *trans*-membrane protein of 572 amino acids that has an integrin and a heparin binding motif, an endosomal sorting signal in the cytoplasmic domain, and a polycystic kidney disease domain of unknown function. It is localized to the cell surface and phagosomal membranes. There is also a secreted variant of the protein that results from ectodomain shedding following cleavage by the metalloprotease ADAM10, such that the cleaved extracellular domain circulates as an apparently biologically active fragment (Hoashi, Sato *et al.* 2010, Rose, Annis *et al.* 2010). Of note, PKC and Ca (2+) intracellular signaling pathways regulate ectodomain shedding from the largely Golgi-modified form of GPNMB in melanocytes (Hoashi, Sato *et al.* 2010). Ectodomain fragments of GPNMB act as a growth factor to induce matrix metalloprotease-3 (MMP-3) expression via the ERK pathway in fibroblasts in C2C12 myoblast cultures (Furochi *et al.* 2007a). The GPNMB ectodomain, released following ADAM10 cleavage of GPNMB from the surface of breast cancer cells, is capable of inducing endothelial cell migration (Rose, Annis *et al.* 2010). Our observations of increased GPNMB levels in the heart associated with decreased circulating GPNMB levels likely represent changes of GPNMB processing involving cleavage or binding in the setting of HF.

Transcriptome data in the HMDP showed that transcript levels of GPNMB and ADAM9 were positively correlated (r = 0.22, p-value = 0.029). Inhibition of ADAM9, a sheddase of ADAM10, reduced the amount of ADAM10 enzyme in the medium while increasing membrane-bound ADAM10 (Moss, Powell *et al.* 2011). We postulate that recruitment of GPNMB-expressing monocytes to the heart occurs along with elevated levels of ADAM9, leading to increased ADAM10 shedding and decreased active ADAM10 at the cell surface, thereby decreasing GPNMB cleavage by membrane-bound ADAM10 and lowering circulatory levels of GPNMB. Alternatively, the endosomal regulation of GPNMB by PKC and Ca (2+) intracellular signaling pathways may determine cell surface expression, ectodomain shedding and circulating levels of GPNMB. Additional studies will be required to fully understand the directionally opposite changes in biomarker abundance in tissue *vs.* plasma.

Due to random selection rather than matched selection, our human controls were not properly matched to the heart failure cohort by demographics and comorbidities. Therefore, we cannot conclude based on our human data alone that lower GPNMB levels are an independent heart failure risk factor. However, our experiments in mice, using matched littermate controls, supports our claim that GPNMB may be a useful independent heart failure biomarker. Finally, we found that GPNMB levels in plasma were independent of proBNP levels, suggesting that GPNMB may be predictive of outcomes based on properties that are dissimilar to the most commonly used biomarker for HF. This characteristic of GPNMB may add a prognostic value to existing clinical practice and, therefore, warrant confirmatory investigation in a larger human cohort. Additional biomarkers that ascertain various properties of HF may be important additions to the full evaluation of HF susceptibility.

In conclusion, we report a proof of concept study illustrating the application of systems genetics data for the identification of biomarkers for HF. We have identified GPNMB as a promising novel plasma biomarker for heart failure based on our preliminary data in two HF mouse models and in human samples. The molecular mechanisms for which GPNMB are implicated in HF warrant further investigation. Additional candidate biomarkers await full characterization toward the goal of distinguishing disease manifestation and progression, precise risk assessment and tailored therapy.
